# Frequency of *Entamoeba* Complex in Individuals Referred to the Medical Laboratories in Jahrom City, South of Iran

**DOI:** 10.1155/2023/8914563

**Published:** 2023-07-10

**Authors:** Zahra Kargar Jahromi, Ali Taghipour, Kavous Solhjoo, Rahim Raoufi

**Affiliations:** ^1^Zoonoses Research Center, Jahrom University of Medical Sciences, Jahrom, Iran; ^2^Department of Medical Parasitology and Mycology, School of Medicine, Jahrom University of Medical Sciences, Jahrom, Iran

## Abstract

This study aimed to determine the prevalence of *Entamoeba histolytica*, *Entamoeba dispar*, and *Entamoeba moshkovskii*, using microscopic and molecular methods in Jahrom city (Fars Province), south of Iran. Stool samples were collected from 360 outpatients referred to the medical laboratories in Jahrom city. Standard parasitological methods, including direct wet mount examination, formalin–ether sedimentation technique, and trichrome staining techniques, were used for detection of *Entamoeba* complex. Nested polymerase chain reaction (PCR) was used to identify the genus/species of *Entamoeba*. *Entamoeba* complex cysts were detected in 2.5% (9/360) of samples by microscopic methods, while 11 isolates were considered positive for the *Entamoeba* complex by nested PCR. Among them, 2 (18.18%) out of 11 samples were *E. histolytica*, 5 (45.45%) were *E. dispar*, and 4 (36.36%) were *E. moshkovskii*. Molecular positivity was more prevalent among females (4.16%), people living in rural areas (3.44%), and people over 60 years old (13.33%). Considering the clinical manifestations, the *Entamoeba* complex infection in patients with fever (7.69%), severe diarrhea (5.10%), and nausea (5%) was high. This study reported the presence of *E. histolytica*, *E. dispar*, and *E. moshkovskii* in Jahrom city. Therefore, we suggest more public health interventions in Jahrom city.

## 1. Introduction

The *Entamoeba* complex includes six species (*Entamoeba histolytica*, *Entamoeba dispar*, *Entamoeba moshkovskii*, *Entamoeba polecki*, *Entamoeba coli*, and *Entamoeba hartmanni*) that reside in the human intestinal lumen [1]. Among them, *E. histolytica*, *E. dispar*, and *E. moshkovskii* are morphologically identical, but pathogenicity of *E. dispar* and *E. moshkovskii* remains obscure [2]. *E. histolytica* is an invasive enteric parasite and remains one of the top three parasitic causes of morbidity and mortality in humans [3]. *Entamoeba* complex is transmitted by the ingestion of amebic cysts via fecal–oral route, usually through contaminated food, water, and vegetables [4, 5].

Although most cases of amebiasis are asymptomatic [6, 7], approximately, 50 million people become symptomatic, with nearly 100,000 deaths annually [3, 8, 9]. Some researchers believe that species, parasite strains, severity of infection, and host conditions may play a role in the pathogenesis and exacerbation of clinical symptoms [6, 7]. Amebic dysentery is a severe form of amebiasis with frequent watery and bloody stools with stomach cramping [10]. On the other hand, fulminant necrotizing amoebic colitis is a very rare complication, which can destroy bowel tissue and lead to bowel perforation and eventual fatal peritonitis [11].

Molecular studies have shown that the prevalence of *E. dispar* is 10 times higher than that of *E. histolytica* worldwide [12, 13]. Also, precise differentiation and identification of the pathogenic and non-pathogenic species and strains would be of primary importance from a therapeutic point of view [14, 15]. Therefore, we designed and implemented the present study to assess the prevalence of *E. histolytica*, *E. dispar*, and *E. moshkovskii* in Jahrom city, south of Iran, using microscopic and nested polymerase chain reaction (PCR) techniques.

## 2. Methods

### 2.1. Ethical Considerations, Study Setting, and Sampling

To conduction of this study, ethical license was achieved by the Ethical Committee of the Jahrom University of Medical Sciences (IR.JUMS.REC.1394.090). In this regard, the informed consent form was signed by the participants. The present cross-sectional study was implemented from March 2015 to January 2016 on 360 persons, who attended the medical laboratories in Jahrom city, Fars Province, Iran. After collecting fecal samples and completing the questionnaires, the samples were immediately transferred to the Research Laboratory of the Department of Parasitology and Mycology (School of Medicine, Jahrom University of Medical Sciences, Fars Province, Iran) for daily microscopic examinations. Considering the criteria, subjects with a history of treatment with anti-parasitic drugs in the preceding 3 months were excluded. A questionnaire containing information such as the age, sex, living area (urban or rural), and clinical manifestations was completed for each individual.

### 2.2. Microscopic Examination

All fecal samples were surveyed to detect *Entamoeba* cysts or trophozoites, using direct wet mount examination, formalin–ether sedimentation technique, and trichrome staining under microscopic observation (Zeiss, Germany, 40× and 100× magnifications). Then, all samples were kept in 70% alcohol at 4°C for DNA extraction and molecular analysis.

### 2.3. Molecular Examination

For this purpose, DNA was extracted from 200 mg of all fecal samples using the manual phenol–chloroform DNA extraction method according to the protocol [16]. The extracted DNA was preserved at −20°C until PCR. In order to detect *E. histolytica*, *E. dispar*, and *E. moshkovskii* from fecal samples, nested PCR was performed using previously described primers and conditions for these parasites [17, 18]. Product sizes for *E. histolytica*, *E. dispar*, and *E. moshkovskii* were determined as 439, 174, and 553 bp, respectively [17, 18]. The PCR amplification reaction was applied in a total volume of 25 *μ*l, containing 12.5 *μ*l of 2× PCR kit master mix (Ampliqon, Denmark), 0.5 *μ*l of each species-specific primer (25 *ρ*mol each), and 1.5 *μ*l of the extracted DNA [18]. Then, the PCR mix was subjected to an initial denaturation at 96°C (2 minutes), followed by 30 cycles—each consisting of denaturation at 92°C (60 seconds), annealing at 56°C (60 seconds), and extension at 72°C (90 seconds). In the following, one cycle of extension at 72°C for 7 minutes was accomplished [18]. One microliter of the first PCR product was used as a template in the nested (species specific) PCR reaction. The nested PCR was performed as described above, except, the annealing temperature was changed to 52°C. All other parameters of the amplification cycle remained unchanged. Finally, 3.5 *μ*l of the amplification product was separated by electrophoresis through 1.8% agarose gel containing ethidium bromide for 45 minutes and was visualized under UV light for bands of DNA of appropriate sizes.

## 3. Results

The studied participants comprised of males (53.4%; 192/360) and females (46.7%; 168/360). As shown in Table 1, participants were divided into eight age groups of 45 people. Among the participants, 203 participants (56.4%) lived in rural regions, while the remaining (43.6%) lived in urban areas. Based on clinical manifestations (Table 2), most patients had flatulence (83.05%) and abdominal cramps (70.55%).

Using microscopic methods, the *Entamoeba* complex cysts were detected in 2.5% (9/360) of the isolates. Based on the nested PCR, 11 isolates were considered positive for the *Entamoeba* complex (Supplementary Figure S1). Among them, 2 (18.18%) out of 11 samples were *E. histolytica* (Supplementary Figure S2), 5 (45.45%) were *E. dispar* (Supplementary Figure S3), and 4 (36.36%) were *E. moshkovskii* (Supplementary Figure S4).

Molecular positivity was more prevalent among females: 4.16% (8/192); people living in rural areas: 3.44% (7/203); and people over 60 years old: 13.33% (6/45) (Table 1). Considering the clinical manifestations (Table 2), the *Entamoeba* complex infection in patients with fever (7.69%), severe diarrhea (5.10%), and nausea (5%) was high. َHowever, the frequency of *Entamoeba* complex was not significantly different in symptomatic and asymptomatic groups (Table 2).

## 4. Discussion

To the best of our knowledge, this study was the first to detect *Entamoeba* complex and differentiation of *E. histolytica*, *E. dispar*, and *E. moshkovskii* in Jahrom city in south of Iran. Furthermore, we described the frequency of *Entamoeba* species in patients with clinical symptoms. In the medical laboratories of many countries, diagnosis of *Entamoeba* is based on the microscopic identification of cysts or trophozoites. Microscopic techniques are often accompanied by misdiagnosis, and it is impossible to differentiate between the isolates of *Entamoeba* complex [19]. Thus, the main purpose of molecular approaches is to differentiate and detect *Entamoeba* species in fecal samples. The results of molecular studies indicate the presence of *E. histolytica*, *E. dispar*, and *E. moshkovskii* infections in the study area. Nonetheless, parallel to other regions of Iran, the prevalence of these amoebae and other parasites has strikingly diminished in recent years [20]. According to the World Health Organization (WHO) report and many local studies, the prevalence of *E. dispar* is higher than that of *E. histolytica* and *E. moshkovskii* [20, 21]. The present findings revealed that 45.45% (5/11) of the samples were attributed to *E. dispar*. The prevalence of *E. dispar* in this study is close to most previous studies conducted in northern, central, and southern Iran [20]. Although the pathogenicity of *E. dispar* and its mechanism of action remains unclear [22, 23], it is suggested that further studies at the cellular level be performed on this amoeba to gain a deeper understanding of its interaction with the host. However, most cases of *E. histolytica* may actually be *E. dispar*, the morphological similarity of which makes them difficult to estimate accurately [24]. Also, it should be noted that most cases of *E. histolytica* infection are reported in asymptomatic patients [7, 25]. A meta-analysis study in Iran showed that 91% (95% CI 80–99%) and 7% (95% CI 0–19%) of amoebae isolated from general population belong to *E. dispar* and *E. histolytica*, respectively, while prevalence of these in patients with gastrointestinal disorders were 75% (95% CI 45–96%) and 18% (95% CI 1–43%), respectively [20].

In the case of *E. moshkovskii*, it was initially assumed to be a non-virulent and free-living species of *Entamoeba* [26]. In four studies from Australia, Tunisia, Malaysia, and Bangladesh, it is shown that humans can be true hosts for this species [1, 27–29]. Therefore, it is recommended that further investigations are necessary to determine the relationship between *E. moshkovskii* and gastrointestinal disorders and to identify the possible pathogenicity of this amoeba. However, the gastrointestinal symptoms of the present studies cannot be fully attributed to these amoebae because other possible factors such as bacterial, fungal and viral infections, or other non-infectious diseases associated with gastroenteritis symptoms have not been investigated and cannot be ruled out. Therefore, we cannot confirm the association between clinical symptoms and *Entamoeba* complex infection, and future research in this area is essential.

As regards risk factors, the present study showed that *Entamoeba* complex was higher among participants aged 60 < years and female participants. This high frequency could be explained by a higher exposure of adult females to the sources of infections as a result of their daily activities such as more frequent consumption of contaminated outdoor food, contaminated water sources, and contact with infected individuals.

In summary, this study reported the presence of *E. histolytica*, *E. dispar*, and *E. moshkovskii* in Jahrom city, especially among patients with gastrointestinal disorders. Based on the findings, the prevalence of *E. dispar* is higher than *E. histolytica* and *E. moshkovskii*. Therefore, we suggest more public health interventions in Jahrom city.

## Figures and Tables

**Table 1:** Frequency distribution of *Entamoeba* complex based on age group, gender, and location by molecular detection.

Age range	Sample size (*n*)	*Entamoeba moshkovskii* (*n*)				*Entamoeba dispar* (*n*)				*Entamoeba histolytica* (*n*)			
		Male		Female		Male		Female		Male		Female	
26–30	45	Rural	Urban	Rural	Urban	Rural	Urban	Rural	Urban	Rural	Urban	Rural	Urban
31–35	45	0	0	0	0	0	0	0	0	0	0	0	0
36–40	45	0	0	0	0	0	0	0	0	0	0	0	0
41–45	45	0	0	0	1	0	0	0	0	0	0	0	0
46–50	45	0	0	0	0	0	0	0	0	0	0	0	0
51–55	45	0	0	0	0	0	0	1	0	0	0	0	0
56–60	45	0	0	0	0	0	0	2	0	0	0	0	1
60<	45	1	0	0	2	2	0	0	0	0	0	1	0
Total	360	4				5				2			

**Table 2:** Clinical symptoms associated with *Entamoeba* complex among participating patients in southern Iran by molecular detection.

Clinical symptom		No. of analyzed sample	No. of positive	*p*-value
Flatulence	No	61	3 (4.91%)	0.37
	Yes	299	8 (2.67%)	
Abdominal cramps	No	106	1 (0.94%)	0.14
	Yes	254	10 (3.93%)	
Mild diarrhea	No	152	7 (4.60%)	0.15
	Yes	208	4 (1.92%)	
Severe diarrhea	No	223	4 (1.79%)	0.08
	Yes	137	7 (5.10%)	
Nausea	No	340	10 (2.94%)	0.61
	Yes	20	1 (5%)	
Fever	No	247	10 (4.04%)	0.54
	Yes	13	1 (7.69%)	
Constipation	No	344	10 (2.90%)	0.46
	Yes	16	1 (3.44%)	

Statistically significant difference at *p* < 0.05.

## Data Availability

All data during study are included in this manuscript and Supplementary Files.

## Supplementary Materials

Supplementary Materials Supplementary description: Supplementary Figure 1. PCR-product of *Entamoeba* complex based on band size (600 bp) by the *Entamoeba* genus-F and -R primer pairs: C−, negative control; 1-11, positive samples. Supplementary Figure 2. PCR-product of *Entamoeba histolytica* based on band size (439 bp) by the *E. histolytica* genus-F and -R primer pairs: 2, negative control; 3, positive control; 4 and 5, positive samples. Supplementary Figure 3. PCR-product of *Entamoeba dispar* based on band size (174 bp) by the *E. dispar* genus-F and -R primer pairs: C−, negative control; C+, positive control; 1-5, positive samples. Supplementary Figure 4. PCR-product of *Entamoeba moshkovskii* based on band size (553 bp) by the *E. moshkovskii* genus-F and -R primer pairs: C−, negative control; C+, positive control; 1-4, positive samples.
