# Supplementary material for: Frequency of Entamoeba Complex in Individuals Referred to the Medical Laboratories in Jahrom City, South of Iran
Source: J Parasitol Res. 2023 Jul 10;2023:8914563. doi: 10.1155/2023/8914563 (PMC10352532; doi:10.1155/2023/8914563)
Supplement: Supplementary Materials — Supplementary description: Supplementary Figure 1. PCR-product of Entamoeba complex based on band size (600 bp) by the Entamoeba genus-F and -R primer pairs: C−, negative control; 1-11, positive samples. Supplementary Figure 2. PCR-product of Entamoeba histolytica based on band size (439 bp) by the E. histolytica genus-F and -R primer pairs: 2, negative control; 3, positive control; 4 and 5, positive samples. Supplementary Figure 3. PCR-product of Entamoeba dispar based on band size (174 bp) by the E. dispar genus-F and -R primer pairs: C−, negative control; C+, positive control; 1-5, positive samples. Supplementary Figure 4. PCR-product of Entamoeba moshkovskii based on band size (553 bp) by the E. moshkovskii genus-F and -R primer pairs: C−, negative control; C+, positive control; 1-4, positive samples. [file 8914563.f1.docx]

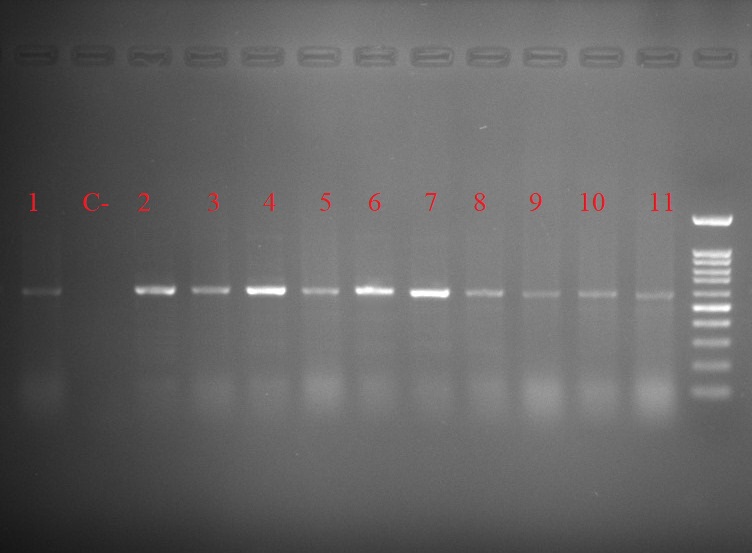


**Supplementary Figure 1.** PCR-product of *Entamoeba* complex based on band size (600 bp) by the *Entamoeba* genus-F and -R primer pairs: C-, negative control; 1-11, positive samples.


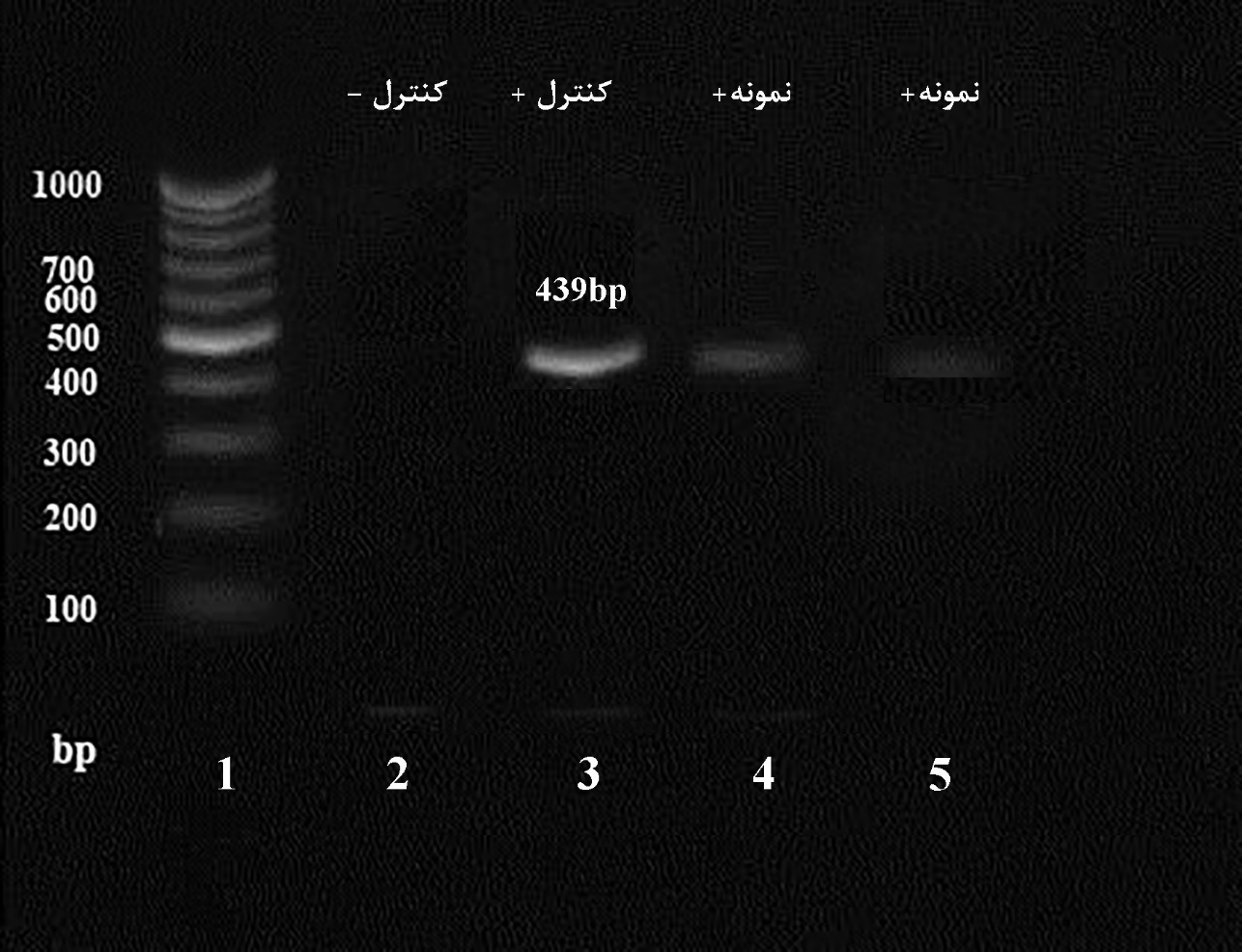


**Supplementary Figure 2.** PCR-product of *Entamoeba histolytica* based on band size (439 bp) by the *E. histolytica* genus-F and -R primer pairs: 2, negative control; 3, positive control; 4 and 5, positive samples.


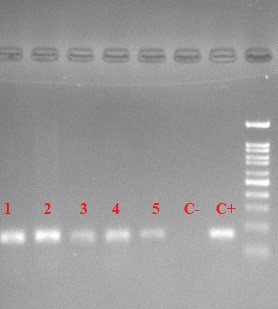


**Supplementary Figure 3.** PCR-product of *Entamoeba dispar* based on band size (174 bp) by the *E. dispar* genus-F and -R primer pairs: C-, negative control; C+, positive control; 1-5, positive samples.


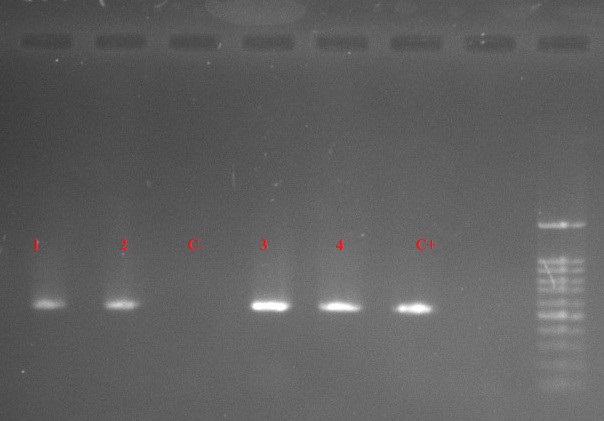


**Supplementary Figure 4.** PCR-product of *Entamoeba moshkovskii* based on band size (553 bp) by the *E. moshkovskii* genus-F and -R primer pairs: C-, negative control; C+, positive control; 1-4, positive samples.
